# Contrasting Transcriptional Responses of a Virulent and an Attenuated Strain of *Mycobacterium tuberculosis* Infecting Macrophages

**DOI:** 10.1371/journal.pone.0011066

**Published:** 2010-06-10

**Authors:** Alice H. Li, Simon J. Waddell, Jason Hinds, Chad A. Malloff, Manjeet Bains, Robert E. Hancock, Wan L. Lam, Philip D. Butcher, Richard W. Stokes

**Affiliations:** 1 Department of Paediatrics, University of British Columbia, Vancouver, Canada; 2 Department of Microbiology and Immunology, University of British Columbia, Vancouver, Canada; 3 Department of Pathology and Laboratory Medicine, University of British Columbia, Vancouver, Canada; 4 Division of Infectious Diseases, British Columbia's Children's Hospital, Vancouver, Canada; 5 Division of Cellular & Molecular Medicine, Department of Medical Microbiology, Centre for Infection, St. George's University of London, London, United Kingdom; University of Delhi, India

## Abstract

**Background:**

H37Rv and H37Ra are well-described laboratory strains of *Mycobacterium tuberculosis* derived from the same parental strain, H37, that show dramatically different pathogenic phenotypes.

**Methodology/Principal Findings:**

In this study, the transcriptomes of the two strains during axenic growth in broth and during intracellular growth within murine bone-marrow macrophages were compared by whole genome expression profiling. We identified and compared adaptations of either strain upon encountering an intracellular environment, and also contrasted the transcriptomes of the two strains while inside macrophages. In the former comparison, both strains induced genes that would facilitate intracellular survival including those involved in mycobactin synthesis and fatty acid metabolism. However, this response was stronger and more extensive for H37Rv than for H37Ra. This was manifested as the differential expression of a greater number of genes and an increased magnitude of expression for these genes in H37Rv. In comparing intracellular transcriptional signatures, fifty genes were found to be differentially expressed between the strains. Of these fifty, twelve were under control of the PhoPR regulon. Further differences between strains included genes whose products were members of the ESAT-6 family of proteins, or were associated with their secretion.

**Conclusions/Significance:**

Along with the recent identification of single nucleotide polymorphisms in H37Ra when compared to H37Rv, our demonstration of differential expression of PhoP-regulated and ESX-1 region-related genes during macrophage infection further highlights the significance of these genes in the attenuation of H37Ra.

## Introduction

*Mycobacterium tuberculosis* (*Mtb*) is a bacterium that can be spread via aerosols generated by infected individuals when they sneeze, cough, or even speak. Inhaled organisms are deposited into alveoli where one outcome is that the bacterium encounters and is ingested by alveolar macrophages [1]. *Mtb* has strategies that enable its survival and replication within the host macrophage, though variations in the pathogenicity of strains have been found [2]. Thus, a comparison of the genomic makeup of a virulent (H37Rv) and an attenuated (H37Ra) strain of *Mtb* should identify virulence genes. Furthermore, comparing gene expression profiles of these strains during their interaction with macrophages should shed light on the genes that enable intracellular survival and the microenvironments encountered during infection.

Here, we have compared the transcriptomes of broth grown and intracellular H37Rv and H37Ra *Mtb*. These two strains were derived from the parental strain H37 *Mtb* that was isolated from the sputum of a patient suffering from chronic pulmonary tuberculosis in 1906 [3]. For most of the next two decades H37 became a widely used laboratory strain of *Mtb* and was shown to be highly virulent for guinea pigs and only moderately so for rabbits – a characteristic described as marking the bacterium as human in type [3]. Subsequently, two daughter strains were identified of which H37Rv maintained its virulence whereas H37Ra did not. However, it should be emphasized that H37Ra is not completely avirulent, only attenuated in its virulence compared to H37Rv. In common animal models for TB such as mice and guinea pigs, H37Ra does establish an infection, and does multiply within these hosts [4], [5], [6].

On the assumption that the attenuated variant must necessarily exhibit alterations to either the genome or the expression of virulence genes compared to the virulent variant, many studies have been conducted to assess differences between *Mtb* H37Ra and H37Rv in order to find mycobacterial virulence factors [7], [8], [9], [10], [11], [12], [13]. Studies comparing the gene expression differences between broth-grown H37Ra and H37Rv or intracellular H37Rv versus broth-grown bacteria have identified differentially expressed genes, some of which have been shown to have roles in mycobacterial virulence [14], [15], [16]. Using microarray technology, Gao *et al*, examined genes involved in the cording and non-cording phenotypes of the respective H37Rv and H37Ra and identified twenty-two genes differentially regulated between them [14]. Using differential display, Kinger and Tyagi examined genes differentially expressed between aerobic cultures of *Mtb* H37Ra and H37Rv and identified the two component system *devR*/*devS* (also known as *dosR*/*dosS*) [15]. This regulatory system has subsequently been observed to aid *Mtb* survival and growth under anoxic conditions [17], [18], [19]. Thus, these studies highlight the importance of the H37Rv/H37Ra model in the search for genes with significant roles in mycobacterial pathogenesis.

Microarray technologies are widely accepted tools used to ask genome-wide questions and numerous studies investigating mycobacterial pathogenesis have successfully utilized these platforms. [19], [20], [21], [22]. However, while there have been microarray studies examining intracellular bacteria [23], [24], [25], none have compared the intracellular transcriptomes of H37Ra and H37Rv, strains that are well documented for their disparities in virulence. Additionally, we sought to examine not just the differences between the two strains inside a host cell, but also the adaptations made by *Mtb* upon encountering a macrophage. For our specific purposes, we chose the bone-marrow-derived macrophage for their reproducibility and similarities to the alveolar macrophage [26], a presumed natural niche for *Mtb* upon infecting the host lung. Previously, our laboratory had used an alternative genome analytical method, bacterial artificial chromosome fingerprint arrays (BACFA), to examine intracellular transcriptomes of *Mtb* [27]. Using BACFA, we were able to identify an operon of genes (*frdABCD*) encoding fumarate reductase, which appeared to be important for intracellular survival of the tubercle bacterium. Thus, we sought to extend our previous findings to compare the gene expression patterns of intracellular H37Rv and H37Ra using whole genome transcriptional profiling to further identify genes that may influence the outcome of mycobacterium-macrophage interactions.

## Materials and Methods

### Ethics Statement

All use of animals was approved by the University of British Columbia Committee on Animal Care in accordance with the Canadian Council on Animal Care recommendations.

### Bacterial Strains

Broth cultures of *Mtb* for RNA extraction were grown in 7H9 liquid medium supplemented with 10% oleic acid, albumin, dextrose complex (OADC) and 0.05% Tween-80. Cultures were grown in 300 mL roller bottles at 3 rpm until mid-logarithmic phase (day 4). For bacteria used to infect macrophages, *Mtb* cultures were grown in Proskauer and Beck media supplemented with 0.05% Tween-80 in 100 mL roller bottle cultures until mid-logarithmic phase. Aliquots were frozen down and thawed as needed for infections.

### Isolation and infection of bone marrow-derived macrophages (BM-MΦ)

BM-MΦ were obtained from the femora, tibiae, and humeri of CD-1 mice as previously described [28], [29]. Monolayers were incubated with a multiplicity of infection (MOI) of 10 bacteria to 1 MФ in binding medium (138 mM NaCl, 8.1 mM Na_2_HPO_4_, 1.5 mM KH_2_PO_4_, 2.7 mM KCl, 0.6 mM CaCl_2_, 1.0 mM MgCl_2_ and 5.5 mM D-glucose) for four hours resulting in an average uptake of approximately 0.1 bacteria per MΦ. Following this incubation, the monolayers were washed three times with pre-warmed media to rinse off unbound bacteria. One group of coverslips were immediately processed and the others were submerged again in cRPMI (RPMI 1640 with 10% FCS, 2 mM L-glutamate, and 1 mM sodium pyruvate) and incubated at 37°C. The remaining groups were processed at days 4 and 7 post-infection; for these groups, both cover slips and supernatants were processed in order to include all viable bacteria within the closed system [30]. Samples to be processed were briefly sonicated (10 s using a VC50T 50 W+3 mm probe (Sonics & Materials, Danbury, CT) tuned as per manufacturer's recommendations) to release and disperse intracellular bacteria, and plated on 7H10 agar plates supplemented with 10% OADC to determine colony forming units (CFU) per well. For all macrophage experiments, three replicate cover slips were assessed at each time point for each of three independent experiments. Statistical significance of comparisons between H37Ra and H37Rv or between two time points was determined using a two-tailed, unpaired Student's *t* test. *P* values <0.05 were marked as significant.

### Infection of MФ for intracellular bacteria RNA extraction

3×10^7^ macrophages were seeded into a 150 cm^2^ tissue culture flask and infected at a MOI of 10 bacteria to 1 MФ in binding medium. After a four hour incubation, monolayers were washed three times with pre-warmed media and then submerged in 30 mLs of cRPMI. At day 5, 25 mLs of cRPMI were added to feed the monolayer. At day 7, 50 mLs of 5 M GTC lysis buffer (per litre: 5 M guanidium isothiocyanate, 7 mL beta-mercaptoethanol [14.3 M, MP Biomedicals], 3.5 mL Tween-80, 0.25% sodium lauryl-sulfate, and 25 mM tri-sodium citrate) were added to the contents of each flask (monolayer and medium). Bacteria were recovered by centrifugation, before Trizol extraction. DNase treatment and purification of extracted RNA was performed as previously described [27].

### Comparative Genomic Hybridization

Genomic DNA was extracted from the bacteria using previously published protocols by Belisle *et al* [31]. Two micrograms each of *Mtb* H37Ra and H37Rv genomic DNA were hybridized to TBv2.1.1 *M. tuberculosis* complex whole genome microarray, generated by the Bacterial Microarray Group at St. George's, as previously described [32] DNA extracted from two biological replicates were hybridized in duplicate. The array design is available in BμG@Sbase (accession number: A-BUGS-23; http://bugs.sgul.ac.uk/A-BUGS-23) and also ArrayExpress (accession number: A-BUGS-23).

### Transcriptome Comparison

RNA was extracted and purified from the bacteria using a differential lysis method developed by Mangan *et al*. [33], from both axenic broth cultures of bacteria as well as intracellular bacteria at day 7 post-infection. Microarray hybridizations were conducted as previously described [32]with 4 µg Cy5-labelled cDNA derived from either H37Rv or H37Ra RNA hybridized against 2 µg Cy3-labelled *M. tuberculosis* H37Rv genomic DNA. RNA extracted from three biological replicates of each strain were hybridized in duplicate.

### Microarray data analysis

The hybridized slides were scanned sequentially at 532 nm and 635 nm corresponding to Cy3 and Cy5 excitation maxima using the Affymetrix 428™ Array Scanner (MWG). Comparative spot intensities from the images were calculated using Imagene 5.5 (BioDiscovery), and imported into GeneSpring GX™ 7.2 (Agilent Technologies) for further analysis. The array data were normalized to the 50th percentile of all genes detected to be present on the array and further normalized to the mean of specific samples depending on the comparison *e.g.* H37Rv in broth versus H37Ra in broth. The dataset was filtered to include only genes called present or marginal (>2 fold signal/background in either Cy3 or Cy5 channels) in at least 60% of hybridizations. Significantly differentially expressed genes were identified using a t-test (*P*<0.05) with Benjamani and Hochberg multiple testing correction and further filtered to include genes with >1.5 fold change. For genomic comparisons, H37Rv and H37Ra hybridizations were compared using Arraypipe (http://koch.pathogenomics.ca/cgi-bin/pub/arraypipe.pl). Genes were filtered for 1.5-fold difference and then analyzed via ANOVA (*P*<0.05). The hypergeometric distribution was used to determine if previously published functional categories of genes were significantly enriched in the comparisons. MIAME-compliant raw and fully annotated microarray data has been deposited in BμG@Sbase (accession number: E-BUGS-86; http://bugs.sgul.ac.uk/E-BUGS-86) and also ArrayExpress (accession number: E-BUGS-86).

### Quantitative RT-PCR analysis of selected candidate genes identified by microarray analysis

One to four micrograms of total RNA were reverse transcribed by priming with random hexamers, with the following added to each tube of total RNA: 5 µL 5X SSII buffer, 2.5 µL 0.1 M DTT, 2.3 µL dNTPs (5 mM dA/G/C/TTP), 1.7 µL water, and 2.5 µL Superscript II. Gene specific primers (Table S5) were used with SYBR Green reagent (Finnzymes) to assess expression of candidate genes. qRT-PCR conditions were as follows: 95°C for 10 minutes followed by 35 cycles of 94°C for 30 seconds, 57°C for 20 seconds, and 72°C for 30 seconds. qRT-PCR data is presented as fold difference of expression in H37Ra relative to H37Rv using the 2^−ΔΔCt^ method as described previously [34] with a 71 bp product (henceforth called rrnAP1) from the promoter region of *rrnS* (*Rvnr01*) in *Mtb* [35] and *dnaK* used as normalizing genes. Two normalizing genes were used for each time point as it has been shown that normalizing genes do not necessarily stay constant over time, and that utilizing more than one control gene is required for reliable expression analysis [36]. Neither rrnAP1 nor *dnaK* were significantly differentially expressed, as determined by microarray analysis, in intracellular or H37Rv/Ra comparisons in this study.

## Results

The strains H37Rv and H37Ra of *Mtb* are highly related, and yet, their growth rates inside macrophages and ability to cause disease are significantly different. We, therefore, sought to identify genes involved in pathogenesis by comparing the gene expression patterns of the two strains. Previously published transcriptomic profiles of H37Rv and H37Ra have focused on axenic broth comparisons, or on differences in gene expression between broth grown and intracellular H37Rv. Our study confirms and extends these reports by comparing directly the respective transcriptomes of intracellular H37Rv and H37Ra.

### 1. Contrasting broth grown H37Rv and H37Ra

**Figure 1:**
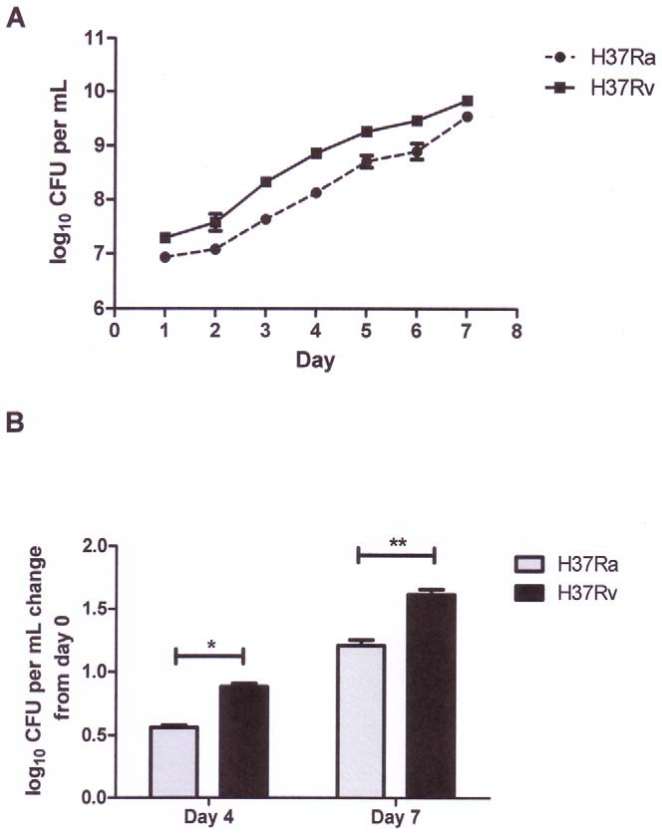
Growth of *Mtb* in 7H9 enriched broth and murine bone-marrow derived macrophages. A) *Mtb* H37Ra and H37Rv were grown in 7H9 enriched media in aerated roller bottle cultures over 7 days. The graph shows the means ± SEM calculated from three independent experiments performed in duplicate. RNA for microarray experiments was extracted from both cultures at day 4. B) BM-MΦ were infected with *Mtb* H37Ra (grey bars) or H37Rv (black bars). Four hours after infection, unbound bacteria were rinsed off and intracellular bacteria were enumerated. Both strains had been internalized equally (ca. 10^4^ bacteria per 10^6^ macrophages). Bacterial CFU at days 4 and 7 post-infection were assessed and are plotted as Log_10_ change from day 0. The means ± SEM calculated from three independent experiments (each performed in triplicate) are shown. Statistical significance was determined using the student's *t-test*: *P = 0.007, **P = 0.0041. *Mtb* RNA was extracted from this macrophage infection model at day 7.

Comparing genomic differences between the strains using CGH microarray analysis confirmed the recently reported sequence similarity [37], [38] along with the RvD2 region of difference present only in H37Ra (Table 1). The growth rate of H37Rv and H37Ra in broth did not differ significantly over the course of seven days (Fig. 1A). In addition, the doubling times calculated at mid-logarithmic phase (4 days) were very similar between strains (H37Ra 22.7±0.7 h, H37Rv 22.2±0.5 h; *P*>0.05). Therefore, gene expression profiling of axenic broth cultures of H37Rv and H37Ra was employed to identify genes that may be constitutively differentially expressed between H37 variants in mid-logarithmic phase (Table S1). Our results correlate well with previous studies that examined cording phenotypes of H37Rv and H37Ra as well as genes under control of the *phoP* regulon. Specifically, we observed significant overlap of genes under-expressed in H37Ra relative to H37Rv with those found by Gao *et al*. [14] (hypergeometric p-value 1.77×10^−3^), as well as genes under the regulation of *phoP* found by Walters *et al*. [39], hypergeometric p-value 1.27×10^−8^). Of particular interest among the 436 genes identified as differentially expressed between the strains were *pks3* and *fadD21*, a *pks3*,*4*-associated fatty acid activating enzyme involved in polyacyltrehalose (PAT) and 2,3-di-O-acyltrehalose (DAT) synthesis [40] that were repressed in H37Ra compared to H37Rv. *pks3* and *pks4* are regulated by *phoP*, and *phoP* mutants in *Mtb* have a deficiency in cord factor synthesis, and an absence of PATs, DATs, and sulpholipids [38], [41].

**Table 1:** Differences revealed in genomic DNA of Mtb H37Ra compared to H37Rv by microarray analysis.

Gene	H37Ra Fold difference compared to H37Rv gDNA ± SEM	Gene description
*Mb1785c*	**2.35±0.33**	Conserved hypothetical protein (unknown function), showing similarity with glycosyl transferases, sulfolipid sulfoquinovosyldiacylglycerol synthases, and hypothetical proteins. No equivalent in *Mycobacterium tuberculosis* strain H37Rv. Belongs to the RvD2 region
*Mb1786*	**7.83±1.34**	Possible sulfite oxidase involved in the degradation of sulphur containing compounds. No equivalent in *Mycobacterium tuberculosis* strain H37Rv. Belongs to the RvD2 region
*Mb1787*	**3.47±0.25**	Probable *mmpL14*, conserved transmembrane transport protein – unknown function, but thought to be involved in fatty acid transport. No equivalent in *Mycobacterium tuberculosis* strain H37Rv. Belongs to the RvD2 region

Genes of the RvD2 region correspond to MRA_1768A and MRA_1768B of the *Mtb* H37Ra genomic sequence, and were absent in H37Rv.

### 2. Intracellular Mtb gene expression signature

To understand the response of H37Rv and H37Ra to an intracellular environment, the transcriptional profile of axenic broth grown bacilli was compared to the respective gene expression pattern derived from bacilli within murine macrophages. During macrophage infection, 81 genes were identified to be significantly induced by both H37Rv and H37Ra, along with 70 that were repressed relative to axenic broth grown bacilli (Table S2). *Mtb* genes induced within the intracellular environment were involved in lipid metabolism (*e.g. fadD33*, *fadE5*, *Rv3229c*, *Rv1344*), intermediary metabolism and respiration (*e.g. Rv1463*, *icl*), transcription regulation (*e.g. Rv1460*, *Rv139*, *sigB*), and response to oxidative stress (*e.g. ahpC*, *ahpD*), These data correlated well with previous reports of *Mtb* gene expression in murine macrophages (Schnappinger *et al*. [23]) (hypergeometric function p = 1.98×10^−17^), or human monocyte-derived macrophage and dendritic cells (Tallieux *et al*. [24]) (hypergeometric function p = 1.97×10^−17^). These commonalities reveal a set of genes, irrespective of macrophage phenotype or bacterial strain, which, presumably enable the bacterium to survive within a host cell.

Genes involved in fatty acid metabolism were induced in intracellular bacteria, particularly by the virulent H37Rv. Whereas a subset of these genes were significantly induced by both intracellular H37Ra and H37Rv (*fadD2*, *13*, *19*, *26*, *33*; *fadE5*, 14, 21, 23, Table S2), the virulent strain induced an additional seven fatty acid-CoA synthases (*fadD15*, *21*, *25*, *28*, *29*, *30*, *31*), an acyl-CoA dehydrogenases (*fadE5*), an enoyl CoA hydratase (*echA6*), and three acetyl CoA transferases (*fadA2*,*3*,4). The upregulation of these genes reflects the utilization of β-oxidation in fatty-acid metabolism and, as suggested previously [23], the induction of multiple genes within these large gene families may point to the requirement of different isoenzymes to catabolize a variety of different fatty acids. However, five of the *fadD* genes (*fadD21*, *26*, *28*, *29*, and *30*) are those previously found to encode for fatty-acyl CoA ligases involved in complex lipid synthesis rather than lipid oxidation, which may reflect the need to maintain or modify the cell wall architecture in a challenging environment [42].

Members of the *mbt* gene cluster (*mbtA*-*J*) which encode for components necessary for mycobactin biogenesis [43] were also differentially represented between intracellular and broth-grown *Mtb*; *mbtB*,*D*,*E*,*F*,*H* and *mbtI* were expressed at higher levels by intracellular bacteria. Mycobactins are mycobacterial siderophores that act as iron chelators [44]. Iron is often limited during infection and microorganisms have developed strategies, such as the production of siderophores, to maximize iron acquisition. The induction of mycobactin synthesis corresponds with the presumably decreased availability of iron inside the phagosome as compared to broth cultures.

### 3. Comparison of intracellular H37Rv and H37Ra profiles reveals differential expression of genes involved in transcriptional regulation and pathogenesis

**Figure 2:**
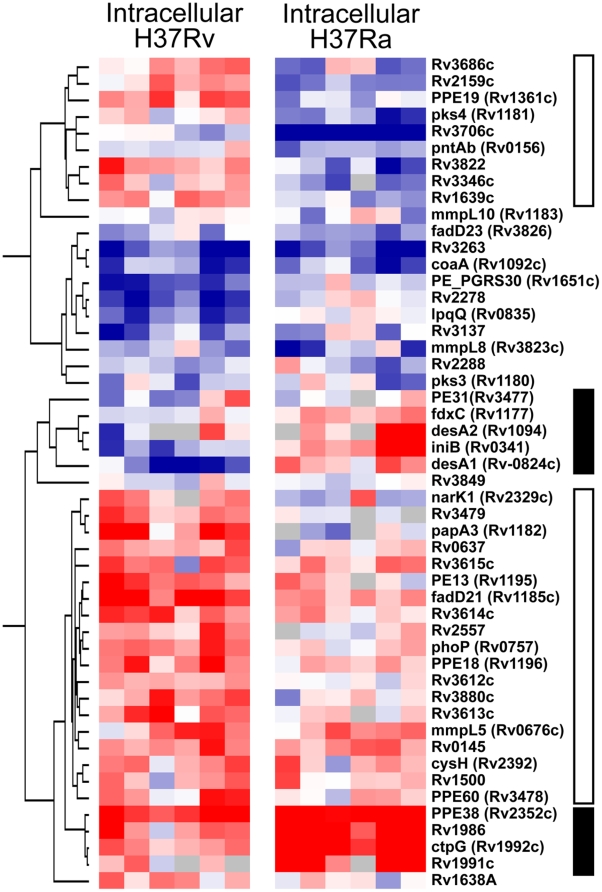
Heat map describing the differences in the intracellular transcriptional signature between H37Rv and H37Ra. Fifty genes differentially expressed between intracellular H37Rv and H37Ra at significant levels are clustered relative to the gene expression pattern derived from each respective broth culture. H37 variants (in 6 replicate hybridizations) are marked as columns, genes as rows. Genes colored in red are induced after macrophage infection compared to respective axenic broth growth; genes colored in blue are repressed. Filled bars mark clusters of genes expressed to a greater level by H37Ra inside macrophages. Open bars mark genes induced intracellularly by H37Rv that are expressed at a lower level by H37Ra inside the macrophage.

**Figure 3:**
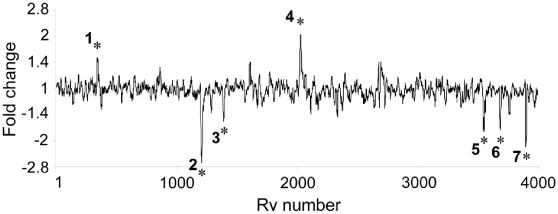
Identification of differentially expressed gene clusters by location mapping. The difference between intracellular transcriptional profiles of H37Ra and H37Rv were plotted onto the chromosome using a moving average. Seven clusters of genes differentially expressed between the two strains inside murine macrophages were identified (marked with asterisks 1–7). Y-axis details differential expression as fold change (H37Ra relative to H37Rv); x-axis describes chromosome location as Rv number. Clusters 1 and 4 were expressed at higher levels intracellularly by H37Ra relative to H37Rv, and clusters 2,3,5,6,7 were expressed at lower levels intracellularly by H37Ra relative to H37Rv. Clusters 2, 4, 5 and 7 contain genes associated with the PhoP regulon, and cluster 6 contains genes (*Rv3614c*-*3616c*) involved in ESAT-6 secretion.

Bacterial growth (Fig. 1B) and the doubling times of intracellular bacteria differed significantly between the two strains at both 4 days (H37Ra 51.5 h±1.7 h, H37Rv 32.8 h±0.9 h; *P* = 0.0007) and 7 days post infection (H37Ra 42.1 h±1.6 h, H37Rv 31.3 h±0.8 h; *P* = 0.0041). Direct comparison of the transcriptional profiles of *Mtb* H37Ra and H37Rv at 7 days post-infection revealed 50 genes that were significantly different in their expression between the two strains (Tables 2 and 3, Figure 2). Of these, 15 genes were over-expressed in H37Ra compared to intracellular H37Rv (Table 2, Figure 2), and 35 genes were under-expressed in the attenuated strain (Table 3, Figure 2). Additionally, the differences in transcriptional response of H37Rv and H37Ra to the intracellular environment were plotted by chromosome position using a moving average to identify clusters of genes differentially expressed between the two strains inside the macrophage (Figure 3, Table S3). This divergence in gene expression not only provides insight into genes that may influence outcomes of infection, but also highlights the polymorphisms uncovered in recent sequence comparisons of H37Rv and H37Ra that probably affect mycobacterial pathogenesis.

**Table 2:** Genes induced in H37Ra relative to H37Rv inside macrophages.

Systematic	Common	Normalized	t-test P-value	Product
*MtH37Rv-0156*	*pntAb*	**1.52**	7.05E-04	PROBABLE NAD(P) TRANSHYDROGENASE (SUBUNIT ALPHA) PNTAB
*MtH37Rv-0341*	*iniB*	**6.88**	1.29E-04	ISONIAZID INDUCTIBLE GENE PROTEIN INIB
*MtH37Rv-0637*	*Rv0637*	**1.83**	3.90E-04	CONSERVED HYPOTHETICAL PROTEIN
*MtH37Rv-0757*	*phoP*	**1.77**	3.04E-04	POSSIBLE TWO COMPONENT SYSTEM RESPONSE TRANSCRIPTIONAL POSITIVE REGULATOR PHOP
*MtH37Rv-0824c*	*desA1*	**2.56**	2.91E-04	PROBABLE ACYL-[ACYL-CARRIER PROTEIN] DESATURASE DESA1
*MtH37Rv-0835*	*lpqQ*	**1.60**	7.76E-04	POSSIBLE LIPOPROTEIN LPQQ
*MtH37Rv-1094*	*desA2*	**2.15**	1.27E-04	POSSIBLE ACYL-[ACYL-CARRIER PROTEIN] DESATURASE DESA2
*MtH37Rv-1177*	*fdxC*	**1.53**	5.34E-05	PROBABLE FERREDOXIN FDXC
*MtH37Rv-1500*	*Rv1500*	**1.85**	8.62E-04	PROBABLE GLYCOSYLTRANSFERASE
*MtH37Rv-1651c*	*PE_PGRS30*	**1.58**	4.68E-04	PE-PGRS FAMILY PROTEIN
*MtH37Rv-1986*	*Rv1986*	**1.90**	6.48E-04	PROBABLE CONSERVED INTEGRAL MEMBRANE PROTEIN
*MtH37Rv-1991c*	*Rv1991c*	**2.57**	5.57E-04	CONSERVED HYPOTHETICAL PROTEIN
*MtH37Rv-1992c*	*ctpG*	**3.27**	4.71E-05	PROBABLE METAL CATION TRANSPORTER P-TYPE ATPASE G CTPG
*MtH37Rv-2278*	*Rv2278*	**1.49**	8.35E-04	PROBABLE TRANSPOSASE
*MtH37Rv-2352c*	*PPE38*	**1.77**	7.83E-04	PPE FAMILY PROTEIN

Genes identified to be significantly expressed (t-test *P*<0.05 with Benjamani and Hochberg multiple testing correction and further filtered to include genes with >1.5 fold change) at a higher level in intracellular H37Ra compared to intracellular H37Rv are listed by chromosome position.

**Table 3:** Genes repressed in H37Ra relative to H37Rv inside macrophages.

Systematic	Common	Normalized	t-test P-value	Product
*MtH37Rv-0145*	*Rv0145*	**0.64**	6.54E-04	CONSERVED HYPOTHETICAL PROTEIN
*MtH37Rv-0676c*	*mmpL5*	**0.64**	2.32E-04	PROBABLE CONSERVED TRANSMEMBRANE TRANSPORT PROTEIN MMPL5
*MtH37Rv-1092c*	*coaA*	**0.61**	8.77E-04	PROBABLE PANTOTHENATE KINASE COAA (PANTOTHENIC ACID KINASE)
*MtH37Rv-1180*	*pks3*	**0.23**	1.00E-05	PROBABLE POLYKETIDE BETA-KETOACYL SYNTHASE PKS3
*MtH37Rv-1181*	*pks4*	**0.23**	1.11E-05	PROBABLE POLYKETIDE BETA-KETOACYL SYNTHASE PKS4
*MtH37Rv-1182*	*papA3*	**0.14**	6.41E-06	PROBABLE CONSERVED POLYKETIDE SYNTHASE ASSOCIATED PROTEIN PAPA3
*MtH37Rv-1183*	*mmpL10*	**0.29**	5.67E-05	PROBABLE CONSERVED TRANSMEMBRANE TRANSPORT PROTEIN MMPL10
*MtH37Rv-1185c*	*fadD21*	**0.26**	6.35E-06	PROBABLE FATTY-ACID--COA LIGASE FADD21
*MtH37Rv-1195*	*PE13*	**0.52**	8.90E-05	PE FAMILY PROTEIN
*MtH37Rv-1196*	*PPE18*	**0.32**	4.98E-06	PPE FAMILY PROTEIN
*MtH37Rv-1361c*	*PPE19*	**0.15**	1.96E-09	PPE FAMILY PROTEIN
*MtH37Rv-1638A*	*Rv1638A*	**0.24**	2.60E-06	CONSERVED HYPOTHETICAL PROTEIN
*MtH37Rv-1639c*	*Rv1639c*	**0.43**	3.23E-04	CONSERVED HYPOTHETICAL MEMBRANE PROTEIN
*MtH37Rv-2159c*	*Rv2159c*	**0.51**	2.64E-06	CONSERVED HYPOTHETICAL PROTEIN
*MtH37Rv-2288*	*Rv2288*	**0.35**	1.20E-05	HYPOTHETICAL PROTEIN
*MtH37Rv-2329c*	*narK1*	**0.36**	1.32E-04	PROBABLE NITRITE EXTRUSION PROTEIN 1 NARK1 (NITRITE FACILITATOR 1)
*MtH37Rv-2392*	*cysH*	**0.51**	7.59E-04	PROBABLE 3′-PHOSPHOADENOSINE 5′-PHOSPHOSULFATE REDUCTASE CYSH
*MtH37Rv-2557*	*Rv2557*	**0.60**	5.41E-04	CONSERVED HYPOTHETICAL PROTEIN
*MtH37Rv-3137*	*Rv3137*	**0.62**	8.75E-04	PROBABLE MONOPHOSPHATASE
*MtH37Rv-3263*	*Rv3263*	**0.58**	3.96E-05	PROBABLE DNA METHYLASE
*MtH37Rv-3346c*	*Rv3346c*	**0.50**	5.48E-06	CONSERVED HYPOTHETICAL PROTEIN
*MtH37Rv-3477*	*PE31*	**0.14**	1.02E-04	PE FAMILY PROTEIN
*MtH37Rv-3478*	*PPE60*	**0.27**	2.33E-04	PE FAMILY PROTEIN
*MtH37Rv-3479*	*Rv3479*	**0.29**	5.42E-04	POSSIBLE TRANSMEMBRANE PROTEIN
*MtH37Rv-3612c*	*Rv3612c*	**0.66**	1.67E-05	CONSERVED HYPOTHETICAL PROTEIN
*MtH37Rv-3613c*	*Rv3613c*	**0.30**	5.09E-05	HYPOTHETICAL PROTEIN
*MtH37Rv-3614c*	*Rv3614c*	**0.28**	1.97E-07	CONSERVED HYPOTHETICAL PROTEIN
*MtH37Rv-3615c*	*Rv3615c*	**0.37**	1.21E-07	CONSERVED HYPOTHETICAL PROTEIN
*MtH37Rv-3686c*	*Rv3686c*	**0.16**	9.67E-05	CONSERVED HYPOTHETICAL PROTEIN
*MtH37Rv-3706c*	*Rv3706c*	**0.58**	8.15E-04	CONSERVED HYPOTHETICAL PROLINE RICH PROTEIN
*MtH37Rv-3822*	*Rv3822*	**0.13**	1.98E-05	CONSERVED HYPOTHETICAL PROTEIN
*MtH37Rv-3823c*	*mmpL8*	**0.28**	4.38E-04	PROBABLE CONSERVED INTEGRAL MEMBRANE TRANSPORT PROTEIN MMPL8
*MtH37Rv-3826*	*fadD23*	**0.60**	6.97E-04	PROBABLE FATTY-ACID-COA LIGASE FADD23
*MtH37Rv-3849*	*Rv3849*	**0.63**	2.16E-04	CONSERVED HYPOTHETICAL PROTEIN
*MtH37Rv-3880c*	*Rv3880c*	**0.60**	6.04E-04	CONSERVED HYPOTHETICAL PROTEIN

Genes identified to be significantly expressed (t-test *P*<0.05 with Benjamani and Hochberg multiple testing correction and further filtered to include genes with >1.5 fold change) at a lower level in intracellular H37Ra compared to intracellular H37Rv are listed by chromosome position.

#### Genes over-expressed in H37Ra indicative of an enhanced stress response

Of the genes induced in the attenuated *Mtb* H37Ra, two were hypothetical proteins (*Rv1991c*, *Rv0637*), one encoded for a component of the isoniazid inducible protein (*iniB,*), and one for a probable ferredoxin (*fdxC*). Both *desA1* and *desA2*, encoding a protein desaturase that is involved in fatty acid biosynthesis and mycolic acid biosynthesis, were also expressed at a higher level in H37Ra compared to H37Rv, suggesting a possible compensatory mechanism to produce desaturated fatty acids to replace lipid moieties lacking in H37Ra [45], [46].

Examining chromosomal gene clusters over-represented in intracellular H37Ra (clusters #1 and #4 – Figure 3, Table S3), we note genes involved in transcriptional regulation (*Rv1990c*, *Rv1994c*) as well as genes induced under antibiotic stress (*iniB*, *iniA*, *iniC*). This suggests that the intracellular environment encountered is more stressful to H37Ra bacilli than it is to H37Rv bacilli 7 days after infection of murine macrophages.

#### Genes involved in ESX-1 secretion are under-expressed in intracellular H37Ra

**Figure 4:**
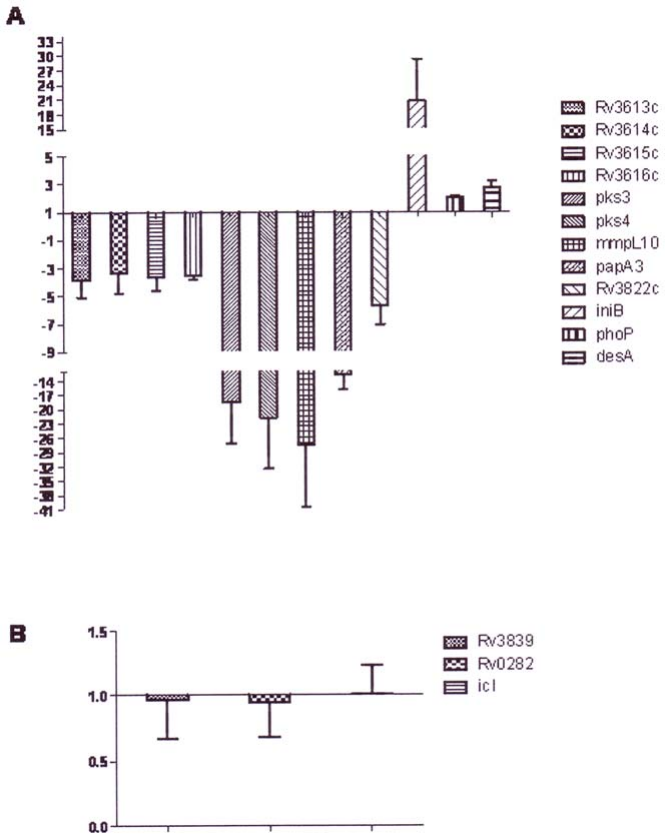
QRT-PCR validation of microarray results for genes differentially expressed between intracellular *Mtb* H37Ra and H37Rv. Quantitative RT-PCR was used to confirm the expression of genes over or under-expressed in intracellular H37Ra compared to intracellular H37Rv. A) Highlighting the *pks3/4 (Rv1180-82)* and ESAT-6-like *(Rv3613-16c)* gene clusters under-expressed in H37Ra compared to H37Rv, and *iniB*, *phoP* and *desA* over-expressed in H37Ra vs H37Rv; and B) genes that were induced by intracellular bacteria, but whose expression did not significantly differ between strains. Data are means ± SEM calculated from three independent biological samples analyzed in triplicate.

One of the gene clusters (cluster #6 Fig. 3), containing adjacent genes *Rv3616c* to *Rv3612c*, was expressed at a lower level in intracellular H37Ra compared to intracellular H37Rv. Two members of which (*Rv3614c* and *Rv3615c*) have been designated a part of the ESX-1 secretion system [47], [48]. Additionally, we also observed a decrease in expression of *Rv3616c* (EspA) in intracellular H37Ra compared to H37Rv by qRT-PCR analysis (Figure 4A). While not in this gene cluster, our microarray data also identified *Rv3849 (espR)*, a recently characterized secreted regulator of ESX-1 [49], as under-expressed in H37Ra. Other genes related to the ESAT-6 family that were expressed at lower levels in intracellular H37Ra compared to H37Rv included PE13 (*Rv1195*), PPE18 (*Rv1196*) and *Rv1198*. It is interesting that PE13 and PPE18 were under-represented along with Rv1198 as it has been previously reported that genes of the ESAT-6 family are often clustered with PE, PPE genes [43], [50]. These ESAT-6 chromosomal arrangements are often flanked by conserved hypothetical proteins, and it has been proposed that such genomic arrangement may encode a secretory apparatus for the ESAT-6-like proteins [43], [50].

#### Members of the *phoP* regulon are differentially expressed between intracellular *Mtb* H37Ra and H37Rv

Twelve members of the *phoP* regulon [39] (*pks3*, *pks4*, *mmpL8*, *mmpL10*, *papA3*, *fadD21*, *PPE18*, *PPE19*, *Rv1639c*, *Rv2376c*, *PE31*, and *PPE60*) were under-expressed in intracellular H37Ra compared to H37Rv. Contrasting these data to a study comparing the transcriptomes of broth grown H37Rv and a *phoP* knockout mutant [39], identified 12 genes regulated by *phoP* that were under-expressed in intracellular H37Ra versus H37Rv (hypergeometric pvalue  = 3.78×10^−31^). Several of these genes are involved in the synthesis of cell envelope constituents that may explain the different lipid profiles between the two strains [51]. In addition, comparing our data to that of Gao *et al*. [14], we found 7 genes implicated in the cording phenotype to be under-expressed in intracellular H37Ra (hypergeometric pvalue  = 6.55×10^−10^).

Taken together, the above results suggest a connection between the ESX-1 secretion system and members of the *phoP* regulon [52]. Combining the fifty differences between the intracellular bacteria with changes in gene expression resulting from the move to an intracellular environment within the macrophage phagosome, *i.e.* examining how the fifty genes differentially expressed intracellularly between H37Ra and H37Rv are regulated inside the macrophage versus axenic broth culture, we are able to delineate which genes virulent strains of *Mtb*, like H37Rv, induce within an intracellular environment. We suggest that this is representative of a successful infection (Figure 2, open bars). The observation that not only does H37Ra over-express only a small subset of these fifty genes (Figure 2, filled bars), but also represses the expression of others, highlights the importance of both ESX-1 and *phoP*-regulated genes during a successful infection by virulent bacilli.

### 4. qRT-PCR validation of microarray data

Expression changes determined by microarray analysis were confirmed by qRT-PCR of three groups of genes: over-expressed (*iniB*, *phoP*, *desA*), or under-expressed (*Rv3822*, *papA3*, *Rv3615c*), in H37Ra when compared to H37Rv (Fig. 4A); and genes induced in intracellular bacteria but unchanged between strains (*Rv3839*, *Rv0282*, *icl*, Fig. 4B). Additional genes were also included in the qPCR analysis to establish correlations between expression of genes that appear to be located in operons on the *Mtb* chromosome. These included members of the PhoP regulon (*pks3*, *pks4*, *mmpL10*), and the cluster of genes (Rv*3613c*-*3616c*) previously characterized to be important for intracellular survival and for interactions with products from the RD1 region [48], [53]. qRT-PCR confirmed the differential regulation of these genes in intracellular H37Ra versus H37Rv (Fig. 4A).

### 5. Transcriptomics and single nucleotide polymorphisms

Recent publication of the genomic sequence of H37Ra identified 57 genes that may be affected by SNPs in H37Ra (compared to H37Rv) [37]. Of these, we found that three were over-expressed in H37Ra compared to H37Rv in an intracellular environment (*phoP*, *Rv0637*, and *PPE38*), and one under-expressed: (*PPE18*). The differential expression of these genes highlights the importance of these 4 genes over the other 53 genes in mediating the attenuated phenotype of H37Ra inside the host. Protein-protein network associations were mapped for these four genes using STRING (string-db.org [54]) in an attempt to identify processes important for intracellular survival that may be affected by SNPs in H37Ra (Figure S1).

PPE18 (Rv1196), under-expressed in H37Ra compared to H37Rv and a member of the PhoPR regulon, has been found to induce interferon-gamma production in infected or previously immunized calves [55]. Examining known protein-protein associations, Rv3616c (MT3718 or EspA) and EspB (Rv3881c) both appear to associate with PPE18. Both EspA and EspB are required for the secretion of ESAT-6 [48], [56]. The expression of *espA* was lower in H37Ra relative to H37Rv in both broth and inside the macrophage, whereas the expression of *espB* differed only between broth-grown bacteria. Interestingly, when modeling PPE38 (induced in intracellular H37Ra over H37Rv), we find associations with several members of the RD1 region (*Rv3875*-*3878*), with *Rv3875* induced in broth-grown H37Ra. PPE38 is not a known member of the PhoPR regulon, and thus, these associations, if proven correct, may indicate a PhoP-independent pathway of ESX-1 secretion.

*Rv0637*, induced in H37Ra compared to H37Rv intracellularly, encodes a subunit of hydroxyacyl-ACP dehydratase with a role in fatty acid synthesis [43]. Examining its protein-protein associations, we find both InhA and Rv0289. InhA is an NADH-dependent enoyl (acyl-carrier protein) reductase whose role in mycolic acid synthesis is well characterized [57]. Rv0289, on the other hand, encodes EspG3, an ESX-1 secretion associated protein [58]. However, neither of these genes shows differences in gene expression. Genes that are both associated with Rv0637 and are induced in broth-grown H37Ra include *Rv0636* (fatty acid synthesis), *rpmG2* (ribosomal protein Rv0634B), and the conserved membrane protein *Rv3587c*.

**Figure 5:**
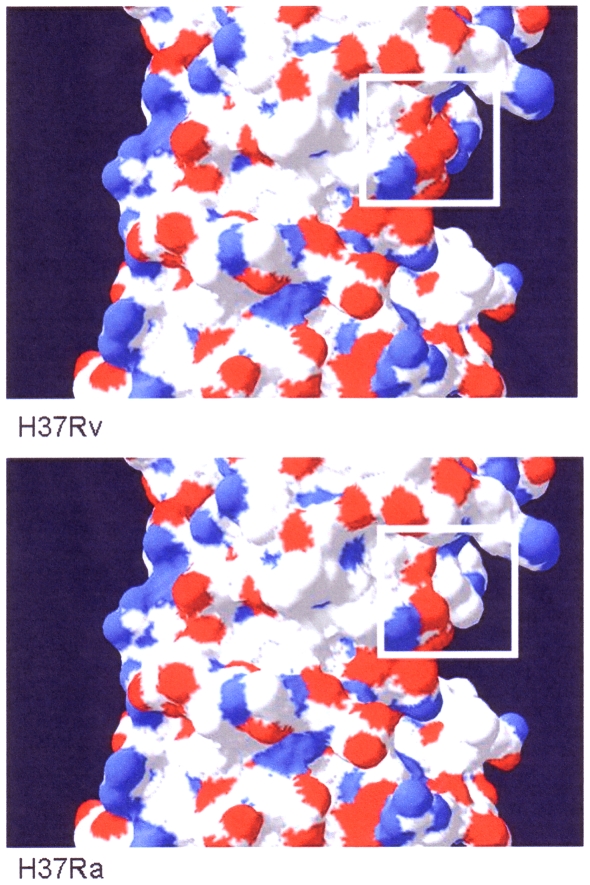
Modeling of PhoP molecule in *Mtb* H37Ra and H37Rv. Using DeepView (Swiss-PDB viewer: http://www.expasy.ch/spdbv/), a 3D depiction of the PhoP molecules from H37Rv (top panel) and H37Ra (bottom panel) were generated including the SNP that results in an amino acid change (from serine to leucine) in the H37Ra PhoP molecule. This substitution is boxed and may result in steric hindrance of binding events with the PhoP molecule.

Lastly, *phoP*, the DNA-binding domain of the PhoPR two-component regulatory system that appears to be involved in bacterial virulence in other organisms [59], [60], was found in this microarray study to be induced in both broth-grown and intracellular H37Ra versus H37Rv (Table 2 and Fig. 4). Protein-association mapping of PhoP leads to several two-component systems (MtrB, TrcS, PrrB, TcrY), one of which (TcrY) was induced in broth-grown H37Ra. These associations suggest that regulation is very finely coordinated between several regulatory systems, and minor changes to one may have profound effects on the cell. Recent genomic sequencing of *Mtb* H37Ra has shown that the sequences of *phoP* in H37Ra and H37Rv differ by one base-pair: cytosine to thymine at position 656. Additionally, it was suggested [38] that this SNP may actually affect the binding of, and regulation by, PhoPR, due to steric hindrance resulting from the change in primary amino acid sequence from serine to leucine at position 219. Sequencing a 200-bp fragment of the *phoP* gene in the H37Ra and H37Rv strains utilized in our laboratory, confirmed this SNP to be present in the H37Ra used to generate the expression data presented here (data not shown). The atomic level mapping of PhoP has been previously published by Wang *et al*. [61] and Gonzalo-Asensio *et al* [62]. We further corroborate these models by using an alternative approach by modeling the differences between H37Ra and H37Rv at the molecular level. Using DeepView (http://www.expasy.ch/spdbv/) to generate three-dimensional models of the PhoP molecules found in the respective strains (Figure 5), the substitution of leucine for serine does appear to change the structure of the protein, and the extra side group may indeed affect binding of PhoP.

## Discussion

This study was initiated to investigate the reasons why an attenuated strain of *Mtb*, H37Ra, grows significantly less well in macrophages than does a related virulent strain, H37Rv. Initially, genomic differences between H37Ra and H37Rv were assessed using microarrays to complement our previous attempts at isolating genomic differences between these strains via two-dimensional gel electrophoresis [63]. Our experiments were in agreement with previous studies that described few major genomic insertion or deletion events between the two strains, other than the RvD2 region of difference. Other differences previously described include restriction site differences, and more recently, base-pair differences [11], [37], [38]. We did not detect point mutations affecting restriction digestion patterns or those identified by genome sequencing [37]. As a single base-pair alteration may not affect annealing of sequences during microarray analysis, these subtle genomic differences were not identified.

The next element of the study surveyed gene expression patterns of the two strains of *Mtb* grown in enriched broth as a means to identify any inherent transcriptional differences between strains. We identified differential expression of *phoP* and genes relating to the cording phenotype [14], which are themselves under the regulation of *phoP* [39]. PhoP is the transcriptional regulator of the two-component system, PhoPR, which is an important regulator of genes with potential roles in mycobacterial virulence.

**Figure 6:**
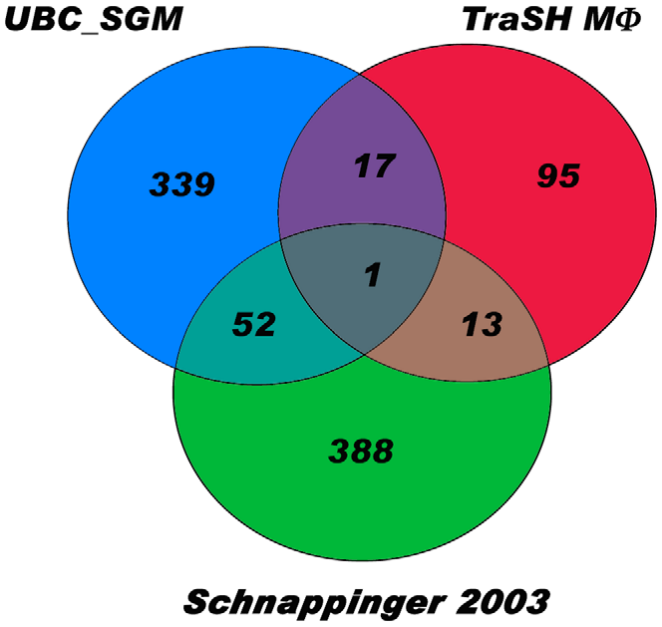
Correlation of datasets examining intracellular expression of *M. tb*. The overlap of genes identified to be of functional importance in studies of the intracellular environment is graphically represented as a Venn diagram using GeneVenn. “UBC-SGM” represents genes induced in *M. tuberculosis* H37Rv identified in this study, “Schnappinger 2003” represents genes induced in intracellular *M. tuberculosis* strain 1254 [23], and “TraSH MΦ” represents genes identified as essential for survival inside BM-MΦ [64].

The transcriptomes of intracellular bacteria, after uptake by murine macrophages, were then compared to their broth-grown equivalents to elucidate differences that may arise due to adaptation to the intracellular compartment. Genes involved in fatty acid metabolism were upregulated by both H37 variants after macrophage infection, along with genes that may protect the bacterium against host defenses. Additionally, genes whose products are involved in aerobic respiration were repressed by intracellular bacteria, probably due to a decreased reliance on aerobic respiratory pathways inside the macrophage. Correspondingly, genes that encode products providing alternative means of biosynthesis or respiration were induced in intracellular bacteria compared to broth-grown bacteria. We compared our data to that of Schnappinger *et al*. [23] that had examined the expression profiles of intracellular versus broth-grown *Mtb* strain 1254. Despite differences between the two studies in the duration of the infection, the bacterial strains used and the growth characteristics of the intracellular bacteria, there was a level of correlation between datasets suggesting that a common set of genes determine a successful infection of a host cell (Figure 6, Table S4). We also compared our data with results obtained using transposon site hybridization, or TraSH, to identify genes important for survival inside murine bone-marrow-derived macrophages [64]. Only one gene, *ahpD*, was common to all 3 studies. *ahpD* encodes an alkylhydroperoxidase which acts in concert with another alkylhydroperioxidase, AhpC to provide antioxidant protection for *Mtb* [65]. Although *ahpC* was in both the Schnappinger *et al*. and our datasets, it was not an absolute requirement for optimal growth inside a macrophage as defined by the TraSH screen [64]. AhpC/AhpD may be important in isoniazid-resistant strains where the catalase-peroxidase KatG has undergone mutation to provide a resistant phenotype [65]. KatG has been ascribed an important role in virulence as it has been found to be required for the catabolism of exogenous peroxides generated by the oxidative burst or peroxinitrites generated by the reaction of superoxide and nitric oxide [66]. Interestingly, *katG* was not found to be induced intracellularly, in either our dataset or that of Schnappinger *et al*. nor was *katG* found to be an essential gene for survival in the macrophage [23], [64]. One possible explanation would be the constitutive expression and subsequent post-translational regulation of *katG*, which would not be detected in a comparative microarray analysis.

With regards to genes commonly upregulated by both intracellular *M. tuberculosis* H37Rv and strain 1254 used in the Schnappinger study, genes involved in fatty acid metabolism (*fadB2*, *fadD19*, *fadD26*, *fadD33*, and *fadE5*), mycobactin synthesis (*mbtB*, *mbtD*-*J*), and [Fe-S] cluster biosynthesis (*Rv1461*, *Rv1462*, *csd*, *Rv1465*) were present in both lists. Additionally, both virulent strains induced a multidrug transporter (*Rv1348*/*Rv1349*) which a recent study suggested is involved in both siderophore synthesis and mycobacterial virulence [67]. Finally, *pckA* which encodes phosphoenolpyruvate carboxykinase (PEPCK) was also seen to be commonly upregulated in these two virulent strains when inside a macrophage. PEPCK catalyses the interconversion of oxaloacetate (OAA) to phosphoenolpyruvate (PEP) and has been thought to be involved in mycobacterial virulence either through its contributions to gluconeogenesis for carbohydrate formation or to the maintenance of the TCA cycle through the conversion of PEP to OAA [68]. Knocking-out *pckA* in *M. bovis* BCG resulted in an attenuated phenotype of the mutant versus wild-type BCG in both aerosol-infected C57BL/6 mice as well as C57BL/6 macrophages [69].

Comparing our list of genes up-regulated in intracellular H37Rv with the list of 126 genes required for optimal growth of H37Rv inside macrophages as defined by TraSH, 18 genes were common between the two datasets. Of this, half were conserved hypothetical genes that have not yet been fully characterized, and others, such as *secA2* [70] and *phoT* [64] have been well studied and are required for growth in both mice and macrophages. *fabG1*, involved in the elongation of fatty acids, has recently been described as an essential gene even under axenic culture conditions [71], [72]. Also present in both the transcriptional and TraSH screens was *clpC*, which encodes for a chaperone protein presumably involved in a varied number of roles including secretion, gene regulation, protein folding and degradation [73], [74], [75]. A recent study characterizing protein-protein interactions in mycobacteria found that ClpC specifically associates with Cfp-10, a component of the ESX-1 secretion system [76]. Such associations will likely further the dissection of secretion mechanisms via the ESX-1 secretion system, and in combination with the requirement of *clpC* for optimal growth, highlight the importance of secretory mechanisms in mycobacterial virulence.

The main focus of this study however, was to compare intracellular expression profiles of H37Ra and H37Rv to identify factors that may explain the differences between the two strains in their pathogenesis and their ability to grow in macrophages. Recent comparisons between H37Ra and H37Rv have identified a base-pair polymorphism in *phoP* [37], [38]. Examining *Mtb* mutants of *phoP*, it has been observed that the PhoPR regulon affects a multitude of cellular adaptation pathways including hypoxic responses (via DevRS), respiration (*nuo* and *ald* operons that maintain NAD+/NADH flux), ESAT-6 secretion (impacting T-cell responses to *Mtb*), antibiotic sensitivity, cellular morphology, and lipid metabolism [39], [52], [77]. These studies not only defined genes that may be under control of the PhoPR regulon, but also demonstrated that one single nucleotide variation (SNV) had profound effects on *Mtb* virulence.

Our study in intracellular bacteria provides further confirmation of the importance of this SNV and its effects on expression of the PhoPR regulon inside the host cell. This single base-pair difference in H37Ra influences the regulation of *phoP* itself with an increase in *phoP* gene expression in H37Ra compared to H37Rv both in broth and inside macrophages. Interestingly, in both axenic culture and intracellularly, genes within the PhoP regulon were expressed at a lower level in H37Ra compared to H37Rv (e.g. *pks3/4*, *fadD21*, *papA3*, *mmpL8*, *mmpL10*). We speculate that the SNV in the *phoP* DNA binding domain of the attenuated strain results in ineffectual expression of the PhoP regulon in H37Ra, and that this in turn is reflected in the compensatory transcriptional induction of the PhoP regulator in H37Ra. Previous studies have described similarities between the lipid profiles (especially the components of PAT, DAT and sulpholipids) of the *phoP* mutants and H37Ra [39], [40], [41] – although it is unclear if the attenuation of the *phoP* mutant is comparable to that of H37Ra. The increased transcript levels of genes involved in PAT, DAT, and sulpholipid synthesis in H37Rv, as seen in our study comparing intracellular H37Ra and H37Rv, are predicted to be a requirement for remodeling of the bacterial cell envelope upon internalization [40]. Additionally, PhoPR involvement in metabolic processes such as fatty acid metabolism has led to the supposition that faulty regulation mediated by this two-component system could also affect bacterial adaptation to host environments in its ability to utilize alternate means of lipid degradation and synthesis. In *Salmonella enterica* Serovar Typhimurium, over-expression of *phoPQ* resulted in an attenuation of bacterial virulence as pathogenesis depended on fine control of transcriptional regulation [60]. Thus, we propose a similar situation where less efficient or unspecific binding of PhoP to regulatory motifs results in the loss of regulatory control of genes involved in cell-wall lipid synthesis resulting in the obvious phenotypic differences between H37Ra and H37Rv, and, additionally, as part of a compensatory mechanism, the over-expression of PhoP by H37Ra.

The difference in *phoP* regulon expression, however, may not be the sole effect of the *phoP* SNV or the sole reason for H37Ra attenuation. Modeling protein-protein interactions via STRING DB, we noted several two-component systems that have been observed to interact with PhoPR, including PrrB, the sensor kinase of the PrrA/B two-component system that may be required for environmental adaptations and early intracellular survival. These interactions suggest that several two-component systems, in concert with PhoPR, coordinate mycobacterial pathogenesis.

An additional contributor to the differing virulence phenotypes may be the ESX-1 secretion system. ESX-1 is encoded for by Region of Difference (RD) 1 which consists of the genes Rv3871 to Rv3878 and secretion of ESX-1 appears to be aided by genes upstream of RD1 (*Rv3868*-*Rv3870*) [78], [79]. RD1 was originally identified via subtractive hybridization to be present in wild-type *M. bovis* strains, but not in the vaccine strain BCG [79]. RD-1 is also present in *Mtb H37*, and interestingly, its expression is lower in broth-grown H37Ra compared to broth-grown H37Rv [80]. ESX-1 secretes ESAT-6 and its chaperone, CFP-10. ESAT-6 has been characterized as a secreted protein, and was originally isolated in short term broth culture filtrates of *Mtb* [81]. ESAT-6 is a virulence factor of *Mtb* and is an important target for T-cell responses in mice, humans, cattle and guinea pigs [82], [83], [84], [85]. Furthermore, ESAT-6 has been shown to have a role in cytolysis enabling virulent mycobacteria to spread intercellularly [86]. Mutants lacking the entire ESX-1 secretion system elicit significantly decreased tissue necrosis in the murine lung [87]. In addition to ESAT-6 and CFP-10, gene products encoded by *Rv3614c*-*Rv3616c* interact with and are also secreted via ESX-1 [8], [48]. Given that our studies show the expression of these associated products and an ESAT-6 family member were higher in intracellular H37Rv versus H37Ra, one could hypothesize that the virulent H37Rv exhibits greater secretory activity for an important set of virulence factors. If the secreted proteins do indeed act as effectors modulating host responses to *Mtb*, it could be reasoned that H37Ra, with its lower expression of these putative virulence factors, is unable to sufficiently alter the host activation state and finds itself in a more hostile intracellular compartment compared to H37Rv. Recently, a study examining the ESAT-6 secretion in H37Ra has postulated a model where ESX-1 function is linked to the PhoPR regulon [52]. It was shown that ESAT-6 secretion in H37Ra was deficient compared to H37Rv, and complementation of H37Ra with H37Rv *phoP* partially restored virulence of the attenuated strain in the mouse and macrophage models. The results of our study reveal a pattern of H37Rv and H37Ra gene expression in broth and inside a murine macrophage infection model that is consistent with this hypothesis. Additionally, modeling of protein-networks of other SNVs (*Rv0637*, *PPE18*, and *PPE38*) suggests genes unrelated to the PhoPR regulon that may also contribute to the regulation of ESX-1 secretion (Figure S1). While *PPE18* is described as under regulation of PhoPR, *Rv0637* and *PPE38* have not been shown to be members of the regulon. However, products of both genes appear to interact with proteins encoded by genes found in the RD1 region (Rv3875-Rv3878) and even an ESX-1 secreted protein, EspG3. This suggests an additional regulatory step of ESX-1 secretion that is independent of PhoP.

Previously, our laboratory reported the use of a bacterial artificial chromosome (BAC) based approach, BAC fingerprint array (BACFA), to analyze expression differences between the two highly related *Mtb* strains, H37Ra and H37Rv [27]. The data presented here are complementary to those obtained using BACFA analysis in that both data sets suggest a scenario where H37Rv is better equipped to adapt to intracellular environments. Although these platforms were complementary there were expression differences exclusively identified using BACFA. This discrepancy may be due to the size and representation of the array elements used in the two techniques, however, this remains to be determined experimentally. Regardless, the microarray experiments did not confirm the differential expression of the *frd* operon, as detected by BACFA analysis and confirmed by qRT-PCR. The *frd* operon encodes for fumarate reductase, which in other bacteria, have been found to be involved in anaerobic respiration [88]. Further, our own studies treating *Mtb*-infected macrophages with a putative inhibitor of fumarate reductase resulted in significant reduction of intracellular growth of mycobacteria [7]. Differential expression of the *frd* operon highlights an important advantage of the BACFA technique: the ability to identify differences in operons. Had the genes of the *frd* operon been on different fragments in the fingerprint arrays, the individual signals would have been too weak to have warranted further analysis even though our additional studies indicate that fumarate reductase may have a role in mycobacterial pathogenesis. With microarrays, however, each gene has its own individual element, analyses designed to incorporate chromosomal location (such as that represented in Figure 3) are required to ensure that genes whose expression are biologically, if not numerically, significant are not overlooked.

By directly comparing intracellular transcriptomes of H37Ra and H37Rv, we have gained further insight into significant pathways required for pathogenesis. Previous microarray studies have examined either genomic or axenic broth differences between the two strains, but our study is the first to attempt a comprehensive examination of these strains in an intracellular environment. The attenuation of H37Ra involves a failure to adapt to both changes in the environment and metabolic constraints, evidenced by the lower expression of genes involved in mycobactin synthesis and fatty acid metabolism when the attenuated strain is moved from broth culture to the macrophage. We demonstrate for the first time differential regulation of the *phoP* regulon by both H37Ra and H37Rv inside a host cell. Additionally, we highlight not only the correlation between PhoP regulation and ESX-1 in intracellular mycobacteria, but also, we suggest additional investigation into PhoP-independent pathways of ESX-1 regulation.

Ultimately, the results presented here demonstrate that the virulent H37Rv responds to entering the intracellular environment by inducing a more extensive and vigorous transcriptomic response compared to H37Ra, that includes genes whose products allow it to adjust to metabolic challenges faced within the host. H37Ra on the other hand, initiates a moderate or, in the case of *frd*, delayed transcriptional program that may not be as effective for enabling adaptation to the novel host environment. This inadequate response to changes in the environment may be the result of dysfunctional control of key signaling cascades such as the PhoPR regulon. This attenuation of H37Ra may be further compounded by a failure to moderate the host intracellular milieu via ESX-1 mediated secretion of effectors.

## Supporting Information

Figure S1 Protein-protein network modeling of interactions that may be influenced by SNPs affecting H37Ra compared to H37Rv. We constructed network models for Rv0637, PPE18 (Rv1196) PPE38 (Rv2352c) and PhoP (Rv0757) using String DB (stringdb.org).
(26.10 MB TIF)

Table S1 H37Ra compared to H37Rv in axenic broth culture. Significantly differentially expressed genes were identified using a t-test (p<0.05) with Benjamani and Hochberg multiple testing correction and further filtered to include genes with >1.5 fold change in broth grown H37Ra compared to broth grown H37Rv.
(0.09 MB XLS)

Table S2 Common H37Ra and H37Rv response to the intracellular environment. Significantly differentially expressed genes were identified using a t-test (p<0.05) with Benjamani and Hochberg multiple testing correction and further filtered to include genes with >1.5 fold change in both intracellular H37Ra compared to broth grown H37Ra, and intracellular H37Rv compared to broth grown H37Rv.
(0.05 MB XLS)

Table S3 Gene clusters revealed by chromosome location mapping as differentially expressed intrcellularly in H37Ra compared to H37Rv. The difference between intracellular transcriptional profiles of H37Ra and H37Rv were plotted onto the chromosome using a moving average. Seven clusters of genes differentially expressed between the two strains inside murine macrophages were identified, and are listed below by chromosome position.
(0.05 MB XLS)

Table S4 Genes common to different intracellular expression studies and macrophage TraSH.
(0.02 MB XLS)

Table S5 Primers (5′-3′) used in qPCR validation of microarray results.
(0.02 MB XLS)
