# Supplementary figures and images for: Contrasting Transcriptional Responses of a Virulent and an Attenuated Strain of Mycobacterium tuberculosis Infecting Macrophages
Source: PLoS One. 2010 Jun 10;5(6):e11066. doi: 10.1371/journal.pone.0011066 (PMC2883559; doi:10.1371/journal.pone.0011066)

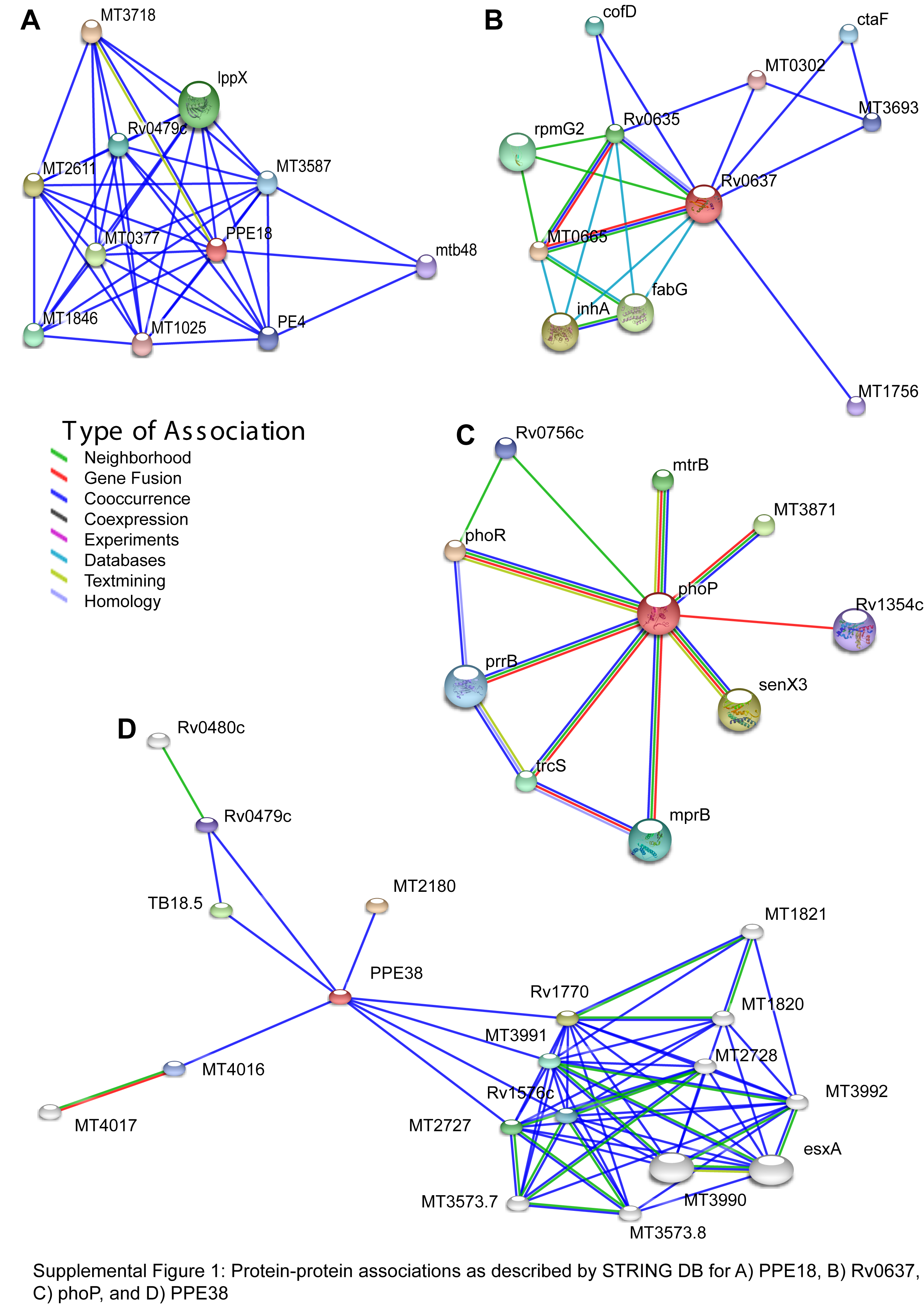

Supplement: Figure S1 — Protein-protein network modeling of interactions that may be influenced by SNPs affecting H37Ra compared to H37Rv. We constructed network models for Rv0637, PPE18 (Rv1196) PPE38 (Rv2352c) and PhoP (Rv0757) using String DB (stringdb.org). (26.10 MB TIF) [file pone.0011066.s001.tif]
